# The Impact of Sea Urchin as an Ingredient on the Physicochemical, Microbiological, and Sensory Properties of Fish Sauce Fermentation

**DOI:** 10.3390/foods12213958

**Published:** 2023-10-29

**Authors:** Mauricio Arango-Herrán, Fini Sánchez-García, Víctor M. Palacios, Ana M. Roldán

**Affiliations:** Department of Chemical Engineering and Food Technology, Faculty of Sciences, University of Cadiz, Puerto Real, 11510 Cadiz, Spain; mauricio.arangoherran@alum.uca.es (M.A.-H.); victor.palacios@uca.es (V.M.P.); ana.roldan@uca.es (A.M.R.)

**Keywords:** fish, sea urchin, fermentation, processing, quality, nutritional, safety, sensory attributes

## Abstract

The consequences of using 25% whole or shelled sea urchin as an ingredient in anchovy sauce on its fermentation and development of its physicochemical properties after 20 days fermentation was studied. Two varieties of fish and sea urchin sauce were made with or without shell at 1:2:1 ratio (salt:fish:sea urchin) plus a control fish sauce at 1:3 ratio (salt:fish). All sauces were fermented at 40–50 °C for 20 days, where for the first 7 days the preparation remained in a static phase. During their fermentation, pH, salt concentration, aw, TVB-N, TMA, total nitrogen, formaldehyde nitrogen, amino nitrogen, and ammonium nitrogen, as well as aerobic mesophiles and lactic acid bacteria were monitored. The fermentation of the experimental sauces proved to follow an evolution rather similar to the control sauce. The whole and shelled sea urchins provided the necessary microbial and enzymatic load to trigger an adequate hydrolysis of the fish and the production of total nitrogen (16.0–17.6 g/L), formaldehyde nitrogen (15.1–16.0 g/L), and amino nitrogen (0.7–0.8 g/L) of the same order as the control sauce, despite the lower fish content. According to TMA (9.2–13.1 mg N/100 g), VBT (40.0–47.2 mg N/100 g) contents, and pH levels (5.41–5.46), no deviation of the fermentation process was observed under the experimental conditions (salt content, temperature, and agitation after the static phase). Quantitative descriptive analysis (QDA) sensory revealed that the use of sea urchin results in high quality products characterized by their aromas of crustaceans and mollusks. The present study investigates the potential use of shelled and even whole sea urchin as an ingredient for the preparation of high quality fish sauces.

## 1. Introduction

Fish sauce is a liquid product obtained by endogenous enzymatic proteolysis from low-value fish or fish-processing waste through the addition of salt and its fermentation under the action of certain halotolerant and halophile microorganisms [1,2]. It is widely used as a food ingredient in most/certain/some Southeast and East Asian countries [3]. Fish sauces, nevertheless, are undoubtedly among the most prevalent products in the cuisine of many countries all over the world [4]. At present, over 300 different types of fermented sauces or pastes are made from fish, shellfish, or mollusks [5]. Nampla (Thailand), budu (Malaysia), bakasang (Indonesia), yu-lu (China), patis (Philippines), shotturu (Japan), nuoc-mam (Vietnam), or ngapi (Myanmar) are some examples of fermented fish products [3,6].

It is traditionally made from a mixture of salt and fish in different proportions that is allowed to ferment [6] in a concrete tank at a temperature range of 35–40 °C, although the fermenting temperature may vary with the different regions [3,7]. During its fermentation, the hydrolysis of the proteins is induced either by the endogenous proteinases that are found in the fish muscle and digestive tract (cathepsins, pepsin, trypsin, chymotrypsin) or by exogenous enzymes from halotolerant microorganisms, which may also play an important role in the process [3,8,9]. As a result of the fish protein hydrolysis, peptides, amino acids, and ammonia are produced with a considerable effect on the sensory characteristics of the final fish sauce [3,6]. In fact, a wide variety of raw materials can be used for the production of fish sauce as long as their proteolytic enzymes content is high enough to achieve the desirable tissue solubilization and protein hydrolysis effects. Fish sauce is commonly made from small size pelagic species like anchovies or sardines [10,11], with the anchovy-based product being the most popular [12]. However, a wide range of sauces are obtained from fermented mollusks (e.g., squid) or shellfish (e.g., shrimp paste or original oyster sauce). In Southeast Asia and Japan, fish sauces are also produced from salmon, shrimp, crab, oyster, sea urchin, or sea cucumber, among others [13,14,15].

Sea urchin gonads are gastronomic delicacies widely appreciated in Europe, principally in different areas of the Mediterranean basin where this product has been largely exploited [16,17]. Thus, around 75,000 tons of sea urchins are commercialized annually worldwide for gonads consumption and this demand has been growing in recent decades [18,19]. Urchin gonads are rich in bioactive compounds such as vitamins, minerals, proteins, fatty acids, and polysaccharides and exhibit anticarcinogenic, anticoagulant/antithrombotic, antimicrobial, and antioxidant properties [20]. However, the yellow-orange gonads, which are the only edible part of an urchin, represent a very small fraction of each specimen, while the rest of the animal is discarded as waste [18]. Several studies have pointed out that the gonads of sea urchins are a good source of high quality protein, long chain omega-3 polyunsaturated fatty acids, micronutrients, sterols, and carotenoids, and they are low in saturated fat [18,20,21]. On the other hand, Matveeva et al. [22] corroborated that urchin gonads are characterized by their high content of essential amino acids such as mainly glutamine, asparagine, glycine, alanine, and arginine, which are responsible for the flavor properties of these products. Moreover, sea urchin roe present high levels of enzymatic activity such as protein proteolysis, lipid hydrolysis, oxidation glycogen, phosphocreatine, ATP, and the formation of melanoidin pigments [23] that substantially enhance the quality of the fish sauces in a short time [22]. Considering both of the aspects above mentioned, i.e., its nutritional characteristics and its enzymatic activity, sea urchin is an ingredient with the potential to improve the nutritional and sensory characteristics of fish sauces and to act as a fermentation activator. Additionally, sea urchin shells were used as fermentation ingredients, as previous studies have shown that residual cell tissues and spines contain polyunsaturated fatty acids, collagen, carotenoids, and small polyphenols [18,19]. The aims of the present study were to assess the influence of sea urchin, including even the shell, as an ingredient in some preparations, in the development of the fermentation, and in the final characteristics of fish sauces ready for consumption.

## 2. Materials and Methods

### 2.1. Raw Materials

The anchovy samples (*Engraulis encrasicolus*) were purchased from a local market in Cadiz (Spain). The sea urchin (*Paracentrotus lividus*) specimens were supplied by the company “Fresco y del Mar, Pesca Gallega Artesanal” located in A Coruña (Galicia, Spain).

### 2.2. Experimental Fish Sauce

The anchovies were used in one piece without beheading or eviscerating, i.e., following traditional fish sauce formulations. A total of approximately 18 kg of anchovies, 18 kg of sea urchins, and 18 kg of salt were used to prepare the different sauce formulations for the study. Formulation 1 was the control sample (C) made up of 75% anchovies and 25% salt. Formulation 2 (SUG) was made up of 50% anchovies, 25% sea urchin gonads, and 25% salt. Formulation 3 (SU) was made up of 50% anchovies, 25% whole sea urchin (the crushed shell was included), and 25% salt. Each formulation was made in duplicate in 5-L fermentation tanks and incubated at 40 ± 5 °C for 20 days. The fish, sea urchin (whole or gonads), and salt were arranged in separate layers in the tanks until full. The preparation was allowed to ferment for 7 days in static mode. From then on, the mixture was stirred every other day. The development of the fermentation process was monitored on days 0, 7, 10, 14, 17, and 20. For this purpose, a sample of the liquid phase was taken and filtered using Whatman No. 1 filter paper. Once filtered, each sample was stored at −80 °C until analysis. Each analysis was performed in duplicate.

### 2.3. Determination of pH and Water Activity (a_w_)

The pH values of the fish sauce samples were determined using a digital pH-meter (Micro pH 2001, Crison Instruments, Barcelona, Spain) equipped with a probe 50 15/50 15T (Crison Instruments, Barcelona, Spain). The pH values were calculated as the mean of the triplicates. The pH-meter was calibrated every 10 measurements using buffers of pH 4.0 and 7.0.

An Aqualab series 3 water activity meter (Decagon Devices, Pullman, WA, USA) calibrated by means of a standard salt solution and fitted with inner temperature control was used to measure the water activity at 25 °C. The samples were placed into disposable sample cups (Decagon Devices Inc., Pullman, WA, USA) ensuring their bottoms were complete cover. The analyses were performed in triplicate.

### 2.4. Determination of Total Volatile Bases (TVB-N) and Trimethylamine (TMA-N) Contents

The content of total volatile base nitrogen (TVB-N) in the fish sauce samples was determined using Conway’s dish method according to the procedure used by Cobb et al. [24] with some specifications described by Sánchez-García et al. [25]. The extract was prepared by mixing 2 mL of the fish sauce samples with 8 mL of 4% TCA in a 50 mL screw cap bottle and was homogenized and stirred for 5 min at room temperature using an ultrasound machine. Afterwards, the solution was filtered and reserved in a glass tube. One Conway’s unit for each sample and one for blank were taken. The reaction reagents were prepared in the cells as described in the method and incubated in an incubator at 45 °C for 45 min. After that, the solution was titrated with 0.02 N HCl until the green colored solution turned to pink. An average titrate volume of HCl was found from the results of three titrations for each sample. The TVB-N content was then determined and expressed as mg/100 g.

The trimethylamine (TMA-N) content of the fish sauce samples was obtained in the same way as TVB-N, with some differences in the addition of reagents, as described in the methodology stated. The concentration of TMA-N was then determined and expressed as mg/100 g.

### 2.5. Protease Activity Measurements

The protease activity, with casein as the substrate, was determined according to the method modified by Kunitz [26]. This method is based on the release of tyrosine that results from the action of protease on casein. In this instance, 1% casein and 0.1 M citrate-phosphate as buffer (pH 5.5) were used. The mixture was prepared in Eppendorf tubes, using 1.5 mL casein, 1.5 mL buffer, and 30 µL sample. This mixture was incubated at 37 °C for 40 min. The reaction was stopped by the addition of 1.5 mL trichloroacetic acid (20%). Next, the tubes were incubated at 4 °C for 15 min and then centrifuged at 12,000 rpm also at 4 °C for 15 min. A blank test was also run for control by adding all the reagents except for the cell-free supernatant. The absorbance was measured at 280 nm by means of a GENESYS™ 10 UV-Vis Spectrophotometer (Waltham, MA, USA). One unit of protease activity was defined as the amount of enzyme that produced a colorimetric response equivalent to 1 µmol of tyrosine per minute at 37 °C.

### 2.6. Determination of the Total Nitrogen Contents

The total N contents were determined using the Kjeldahl method [27]. The 1 g samples were digested in an automated digester, DK6 model (Velp Scientific, Usmate, MB, Italy) and distilled by means of a Kjeldahl Distillation Unit Udk 127 (Velp Scientific, Usmate, MB, Italy). The total nitrogen contents were expressed as percentages.

### 2.7. Determination of Formaldehyde and Amino Nitrogen Contents

The formaldehyde nitrogen contents were determined by titration following the method described by Dissaraphong et al. [28]. Each diluted sample (1:10) was titrated to pH 7.0 using 0.1 N NaOH, and then 10 mL of formalin solution (38%, *v*/*v*; pH 9) was added for neutralization. The titration to pH 9 was continued using 0.1 N NaOH. The nitrogen content in the formaldehyde was expressed in nitrogen g/L.

For the amino nitrogen determination, a 10-fold dilution sample (50 mL) was placed in a 400 mL Kjeldahl flask containing 100 mL distilled water and 3 g MgO. The mixture was distilled and the distillate was taken up in 50 mL of 4% boric acid consisting of the mixed indicator (methyl red:bromocresol green:methylene blue). The solution was then titrated with 0.05 N H_2_SO_4_ to reach the end point. The ammoniacal nitrogen contents were expressed as nitrogen g/L.

### 2.8. Determination of the Salt Content

The salt contents were determined according to Mohr’s titration method with minor modifications [29], where 0.17–0.19 g of fish sauce was diluted in 50 mL distilled water, using potassium chromate (0.1 M) as indicator. 5 mL of the dissolution was titrated against a silver nitrate solution (0.1 M) standardized with commercial sodium chloride (Panreac). The percentage of salt was calculated according to the following equation:

(1) $$Salt\left(\%\right)=\left(\left(\frac{250\;mL}{10\;mL\times 25\;g}\right)\times \left(S-B\right)\times F\times 100\right)$$

where *S* = Titration volume of the sample (mL), *B* = Titration volume of the blank (mL), and *F* = conversion factor of 1 mL 0.1 M AgNO_3_ to 0.005844 g NaCl.

### 2.9. Microbiological Analyses

For the microbiological analyses, 1 mL sample was 10-fold diluted in a 0.1% (*w*/*v*) peptone solution from which serial dilutions were made. The mesophilic aerobic counts were performed using TSA agar plates (Tryptic soy agar with 10% NaCl) incubated at 37 °C for 1–2 days. The lactic acid bacteria (LAB) counts were performed on MRS agar plates (Man, Rogosa, and Sharpe, with 10% NaCl and 50 mg/mL cycloheximide) incubated at 37 °C for 2–3 days. The microbiological counts of the fermented product were expressed as log CFU/mL.

### 2.10. Sensory Evaluation

The resulting sauces were evaluated through a quantitative descriptive analysis (QDA). Six aromatic descriptors (fresh fish, meat, smoked, crustacean, dried/salted fish, and mollusk) and four kinds of flavors (salty, umami, crustacean, and fish) were selected for evaluation by an internal panel of ten members (six women and four men between the ages of 25 and 55). A 5-point intensity scale was used, where “0” indicated an imperceptible attribute and “5” represented the highest level of intensity. In addition, a hedonic scale (dislike very much, dislike slightly, neither like nor dislike, like slightly, like very much) was incorporated into the tasting sheet for the tasters to indicate their preferences.

### 2.11. Statistical Analysis

All the experiments were performed in triplicate, and their average value with the standard deviation was recorded. The statistical analysis was conducted using the analysis of variance on SPSS for Window 19.0 (SPSS Inc., Chicago, IL, USA). A one-way ANOVA test was used to determine the statistical significance (*p* < 0.05) of the sample means.

## 3. Results and Discussion

### 3.1. Evolution of pH

**Figure 1:**
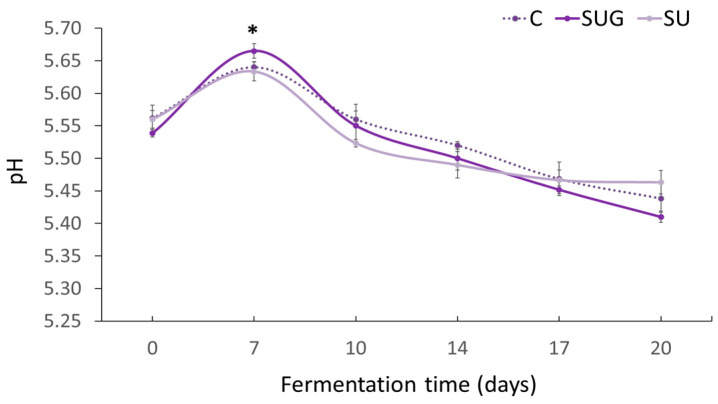
Evolution of the pH level in the fish sauces during the fermentation process. (C: control; SUG: sea urchin gonads; SU: whole sea urchin). Asterisks indicate the significant difference between the control samples (C) and the samples with sea urchin addition (SUG or SU) at each of the sampling time points (*p* < 0.05).

pH is a highly relevant factor that must be taken into account during the fermentation process [7]. According to the Codex Standard [30], the pH of fish sauce should be between 5.0 and 6.5. The evolution of pH levels during the fermentation of all the experimental fish sauces, i.e., the control sauce and the ones which had been added with either whole sea urchin or its gonads are displayed in Figure 1. The initial pH value of all the samples was approximately 5.55 ± 0.10 and no significant differences were observed when comparing the control sample against those where whole sea urchin or its gonads had been used as an ingredient. All of the pH values increased during the first stages to subsequently decrease until the end of the fermentation, when the values had dropped clearly below the initial ones (5.44 ± 0.03). The release of compounds such as organic acids (lactic and acetic acid), ammonia, free amino acids, amino acids of oligopeptides, and free hydrogen ions during the fermentation process resulted in a lower pH of the sauces [7,31,32]. These compounds had been released due to the combined action of the enzymes from the fish flesh and due to the presence of highly salt-tolerant microorganisms [8], with lactic acid bacteria being the predominant microorganisms in numerous fermented fish products [32]. Bacteria consume carbohydrates and reduce the pH of the fermented products as they produce certain organic acids, such as lactic or malic acids [32]. However, the presence of a low number of “spoilage-specific” bacteria, which do not always represent the largest proportion of the total bacterial flora, may produce large amounts of spoilage-related nitrogenous compounds such as TMA and TVB-N that cause an increment of pH levels [3]. This would explain the initial upward trend of pH during the initial stages. After such an initial stage, only those microorganisms that are resistant to the environmental conditions will survive [3,33]. On the one hand, the salt/fish ratio, among other factors, has a considerable influence on the diversity of microbial populations responsible for the development of the fermenting process [8,34].

According to Taira et al. [35], salt/fish ratios equal to or greater than 1/3 result in the predominant formation of organic acids, since these high salt/fish ratios inhibit any massive development of bacteria and any other microorganisms related to the synthesis of volatile bases, such as ammonium or trimethylamine [31,34]. On the other hand, high fermentation temperature levels (40–50 °C) contribute to the inhibition of bacterial development, except for halophilic mesophilic bacteria, which may still develop to a certain level [11,33]. The salt/fish ratios and temperatures used in the present work would explain why the pH levels of the different types of sauces and their evolution did not exhibit any significant differences. Nevertheless, it should be noted that the pH of the whole sea urchin-added sauces did not drop as much as those corresponding to the C and SUG samples, even if their values were rather similar. This is explained by a heavier contribution with microbial loads and a higher enzymatic activity in the SU sauces.

The pH values reached at the end of the fermentation were similar to those determined in commercial fish sauces from Thailand, Vietnam, South Korea, and Japan, i.e., between 5.4 and 5.8 pH [36,37].

### 3.2. Evolution of the Salt Content (%NaCl) and Water Activity (a_w_)

**Figure 2:**
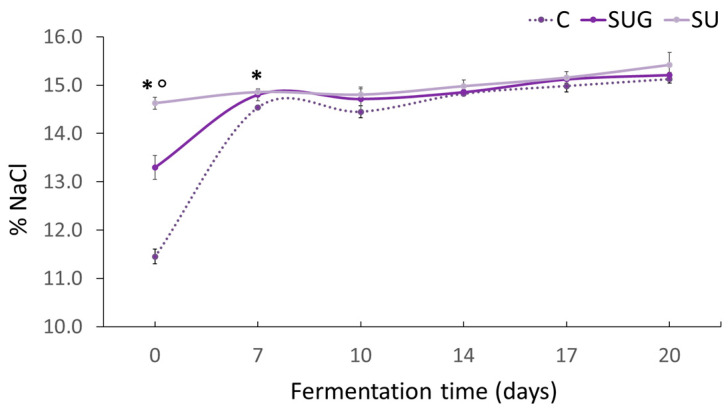
Evolution of the salt content levels (%) in the fish sauces during the fermentation process. (C: control; SUG: sea urchin gonads; SU: whole sea urchin). Asterisks indicate the significant difference between the control samples (C) and the samples with sea urchin addition (SUG or SU) and the symbol ° indicate the significant differences between the sea urchin samples SUG vs. SU at each of the sampling time points (*p* < 0.05).

The evolution of the salt content levels in the fish sauces is shown in Figure 2. It can be seen that at the beginning of the process (t0), the salt content levels of the samples were very different, even though the same proportion of salt had been used in all of the cases. Thus, the control fish sauce showed the lowest value (11.45%), while the highest level (14.62%) corresponded to the sauce where whole sea urchin (SU) had been added. The main variations in the sauce salt content levels took place during the static phase (first seven days), where the diffusion of the salt and the release of exudate fluids depended on the raw materials and the proportions used. The layers of fish and salt produce a pressing that facilitates the osmotic dehydration of the fish [7]. Thus, in the control sauce, where 75% was anchovy with a high water content, the dialysis mechanism and the osmosis between the brine originated and the fish flesh occurred quickly with the consequent water release and sodium chloride diffusion [7,38]. When 25% sea urchin was added, either with or without the shell, and the proportion of anchovies was reduced to 50%, there were lower moisture contents and therefore higher initial solute concentrations, which resulted in a slower osmosis. During the dynamic phase, as agitation facilitated the disintegration of the fish tissues, the contact between the salt and the fish flesh was favored, which in turn favored the diffusion of the NaCl into the tissues and the exudate liquid until saturation.

This resulted in no significant differences between the final salt content levels in the fish sauces at the end of the process (approx. 15%), which is a very similar value to that reported for fish sauces by other authors [39,40].

**Figure 3:**
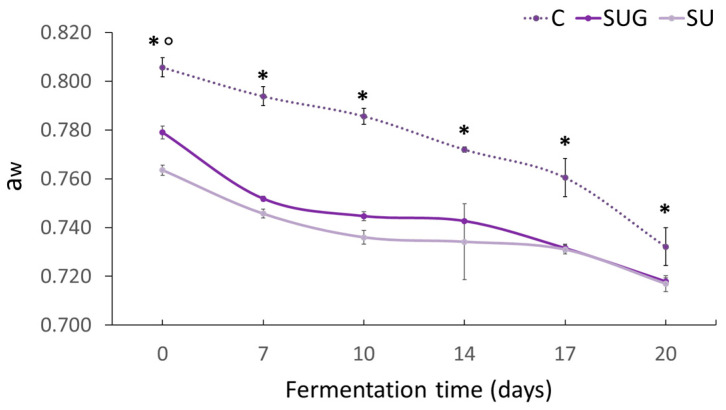
Evolution of a_w_ in the fish sauces during the fermentation process. (C: control; SUG: sea urchin gonads; SU: whole sea urchin). Asterisks indicate the significant difference between the control samples (C) and the samples with sea urchin addition (SUG or SU) and the symbol ° indicates the significant differences between the sea urchin samples SUG vs. SU at each of the sampling time points (*p* < 0.05).

Regarding the water activity (a_w_) (Figure 3), very different values were observed between the initial fish sauces, which seems to be directly related to their NaCl content (Figure 2). The control fish sauce, with a low salt content, exhibited the highest water activity value because of the high release of exudates and poor salt dissolution in the generated brine. The urchin-added sauces presented a lower volume of initial liquid exudates, which resulted in a higher salt concentration in the liquid medium and, therefore, a lower water activity. Salt’s ability to dissolve in water makes it a humectant and, therefore, it absorbs part of the water in the medium (hygroscopic) and reduces water activity (a_w_) [3,41]. Additionally, in the case of whole urchin-added sauces, the composition of the sea urchin shell may include calcified structures made up of calcite, which is rich in highly water-soluble magnesium [42] and may also reduce water activity.

The final water activity levels of the sauces once the fermentation had been completed were similar to those observed in other studies on anchovy sauces, with similar percentages for NaCl (25%). Whereas sardine or bakansang fish sauces, which are made with lower proportions of salt (10% and 8–9% NaCl, respectively), exhibited lower water activity values at a_w_ = 0.860–0.890 [6] and a_w_ = 0.815–0.822 [43], respectively.

### 3.3. Evolution of the Total Volatile Bases (TVB-N) and Trimethylamine (TMA-N) Contents

**Figure 4:**
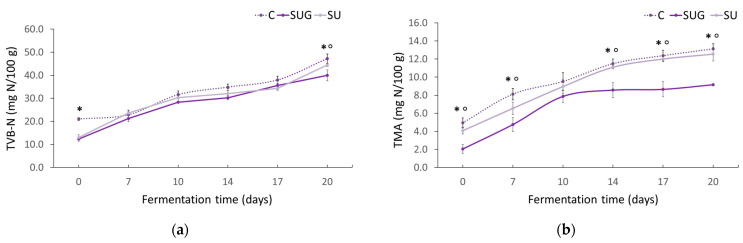
Evolution of the TVB-N (**a**) and TMA (**b**) contents in the fish sauces during their fermentation process. (C: control; SUG: sea urchin gonads; SU: whole sea urchin). Asterisks indicate the significant difference between the control samples (C) and the samples with sea urchin addition (SUG or SU) and the symbol ° indicates the significant differences between the sea urchin samples SUG vs. SU at each of the sampling time points (*p* < 0.05).

The evolution of total volatile bases (TVB-N) and trimethylamine (TMA) contents in the fish sauce samples during their fermentation are shown in Figure 4. These parameters are freshness indicators for unprocessed marine products [44]. According to Kaur et al. [45], TVB-N and TMA-N are found at levels below 35 mg/100 g and 5 mg/100 g, respectively, in fresh seafood. As can be seen in Figure 4a,b, the initial TVB-N and TMA-N contents in the fish sauces were below these levels, which corresponds to good levels of freshness. 

However, the TVB-N initial content (Figure 4a) was dependent on the species included in the formulation and the proportion of these in the mixture. Thus, for the control sauce, which had been made exclusively using anchovies, a higher content of TVB-N was detected (21 mg TVB-N/100 g). Likewise, the differences observed between the initial contents of TMA were due to the species used and to their respective proportions. The increments in TVB-N and TMA-N content levels during the fermentation might be attributable to the activity of endogenous enzymes and bacteria and, subsequently, to the accumulation of trimethylamine, dimethylamine, monoethylamine, ammonia, and other volatile bases [44]. No significant TVB-N content evolution differences could be observed between the three formulations, with levels remaining below the maximum established by the United States Food and Drug Administration (US FDA) for salt-processed fish products (200 mg/100 g) [46].

Regarding the sauces TMA content, significant differences can be observed in Figure 4b between the whole sea urchin-added sauce and the urchin gonads-added sauce. In fact, whole sea urchin-added sauces presented a similar evolution to that of the control sauce, whereas the gonads-added sauce exhibited TMA increments during the first 10 days and then it remained between 8–9 mg TMA-N/100 g. Considering that the internal and external surfaces of marine plants and animals provide an ideal habitat for bacterial communities [47], sea urchin shells may be rich in microorganisms that could promote fish spoilage.

### 3.4. Evolution of the Protease Activity

**Figure 5:**
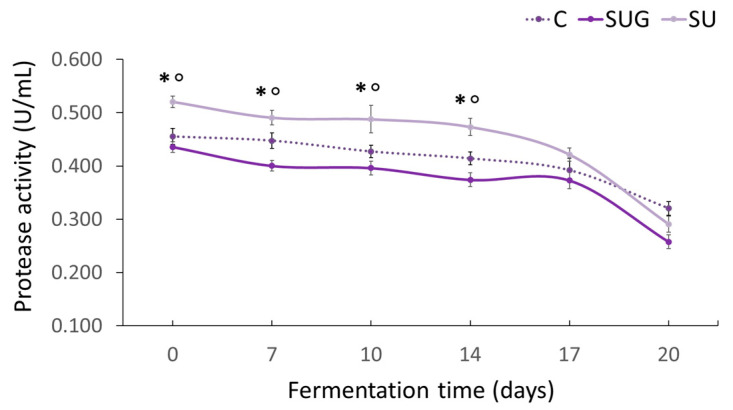
Evolution of the protease activity of the fish sauces during the fermentation process. (C: control; SUG: sea urchin gonads; SU: whole sea urchin). Asterisks indicate the significant difference between the control samples (C) and the samples with sea urchin addition (SUG or SU) and the symbol ° indicates the significant differences between the sea urchin samples SUG vs. SU at each of the sampling time points (*p* < 0.05).

The evolution of the protease activity of all the fish sauce samples during their fermentation is shown in Figure 5. Initially, all three sauce samples showed high protease activity levels, which seems to indicate that sea urchin has a high enzyme activity and/or a high load of protease-producing bacteria. Some authors [48,49] have reported the diversity of digestive enzymes (amylases, lipases, proteases, and cellulases) in sea urchin, highlighting a greater protease activity (especially serine protease chymotrypsin) when compared against other marine species [49,50].

The enzymatic profile of each species also depends on its feeding habits [49] and on the exogenous digestive enzymes of the bacteria present in the ingested food [48]. Thus, Jamaludin et al. [51] determined up to eight isolates of protease-producing bacteria in Bekasang produced through spontaneous fermentation and with the addition of sea urchin gonads. The identification of five of the eight isolates confirmed the presence of *Staphylococcus piscifermentans*, *Staphylococcus saprophyticus*, *Staphylococcus condimenti*, *Bacillus amyloliquefaciens* subsp. *Plantarum*, and *Lactobacillus plantarum* [51].

During the fermentation of the fish sauces, the protease activity slightly decreased over time until day 17, after which there was a significant drop in the protease activity of those sauces containing urchin. The decrease in the protease activity during fermentation, which was more pronounced over the last stages of the process, could be explained by the denaturation of the enzymes as a result of the high temperature (40–50 °C), the salt content increment (Figure 2), and the reduction of a_w_ (Figure 3) and pH (Figure 1). Numerous authors have reported the relevant influence of salt concentration, pH, and temperature [9,49,51,52,53] on the reduction of the protease activity during the fermentation of fish sauces by enzyme denaturation. However, we should also take into consideration that it could as well be attributable to its inhibition by the final product because of its amino acids and short chain peptides content as indicated by [54].

### 3.5. Evolution of the Total Nitrogen, Formaldehyde Nitrogen, and Ammonia Nitrogen Contents

**Figure 6:**
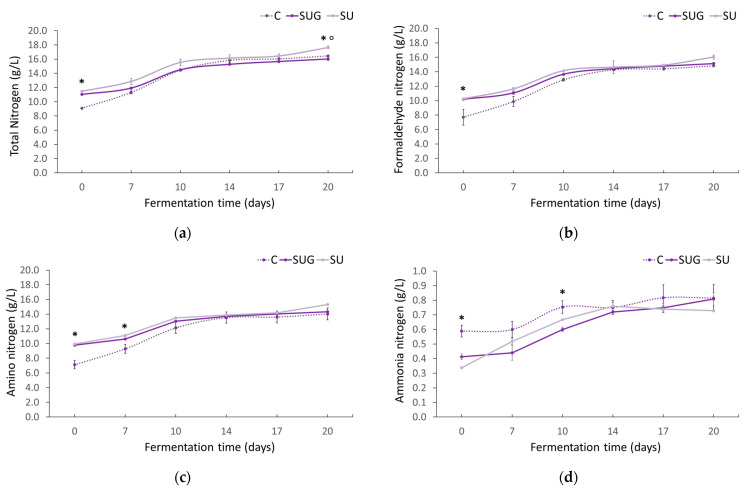
Evolution of the total nitrogen (**a**), formaldehyde nitrogen (**b**), amino nitrogen (**c**), and ammonia nitrogen (**d**) contents in the fish sauces during the fermentation process expressed as g/L (C: control; SUG: sea urchin gonads; SU: whole sea urchin). Asterisks indicate the significant difference between the control samples (C) and the samples with sea urchin addition (SUG or SU) and the symbol ° indicates the significant differences between the sea urchin samples SUG vs. SU at each of the sampling time points (*p* < 0.05).

Figure 6 shows the evolution of the total nitrogen, formaldehyde, amino, and ammonia contents in all the fish sauce samples throughout their fermentation period. The total nitrogen content (Figure 6a) of the fish sauces went up as the fermentation time increased. This is due to the hydrolysis caused by the combined effect of the autolysis and microbial degradation of the fish flesh [1]. This proteolysis is induced by endogenous proteases from the muscles’ tissue and from the digestive tract of both the fish and the sea urchins, as well as by the natural microbiota found on the outer surface of the fish skin and the sea urchin shells. In all of the samples, the total nitrogen increased rapidly over the first 10 days and no remarkable changes could be observed afterwards. This increment in the total nitrogen content during the initial fermentation stage could be related to osmosis [55] and to the mixing and grinding of the ingredients resulting from the agitation of the mixture from the seventh day of fermentation. Xu et al. [53], based on the studies by Lopetcharat et al. [7], reported that the combined effect of the high enzymatic activity and the grinding could lead to a fast fermentation of the fish sauce, as was also observed in this work. 

On the one hand, according to our data, the use of sea urchin (with or without shell) favored the liquefaction of the mixture and gave place to a higher nitrogen content in a shorter time, even when a smaller proportion of fish was used. Furthermore, the use of the shell (SU) further favored the process, which seems to indicate that not only the endogenous and microbial enzymatic contributions from the sea urchin gonads were relevant, but also the microbial contributions from the shell. On the other hand, the final total nitrogen content in the fish sauces has been confirmed to depend on the species of fish used and on the chemical composition of the same [46,52].

As anchovy represented the highest proportion of the species used in the present work, this should limit the amount of final nitrogen in the sauce. However, as can be seen in Figure 6a, the highest nitrogen content was registered in the SU samples, which leads us to think that, in this case, the proteolytic activity contributed by sea urchin and its shell was more relevant regarding the degradation of the fish flesh. Total nitrogen content in the liquid is one of the most important factors with regard to fish sauce quality and, therefore, the regulatory standards for quality grading and pricing are based on this parameter. According to some authors [56,57], high quality fish sauce must have at least 1.5% total nitrogen content, as measured by the Kjeldahl method. The total nitrogen content levels reported for different types of commercial fish sauces produced in Japan, Thailand, China, or Italy are in the range 1.2–2.1% [11,36,57]. The percentages registered for the final sauces produced in the present work were between 1.6 and 1.8%, which could be considered high quality products. These results indicate that, with the raw materials used under the experimental conditions, an efficient protein hydrolysis was achieved in a shorter timeframe than that used for the production of commercial fish sauces.

As can be seen from Figure 6b, the evolution of the formaldehyde nitrogen contents was similar to that of the total nitrogen content, which is in agreement with the reports from other authors [1,58]. Formaldehyde nitrogen is used as a convenient indicator of protein hydrolysis development [59], and it is also used to measure the total free amino acid [60]. An increment of this parameter suggests an increased hydrolysis of the peptides by the endogenous or microbial proteinases [52], which results in a greater free amino group content [60]. No significant differences were observed between the use of sea urchin with (SU) or without its shell (SUG), but fish sauce C showed the lowest formaldehyde content up to day 10 and, therefore, the lowest hydrolysis process. These results corroborate, once again, the substantial influence of sea urchin on the fermentation of fish sauces and the relevance of its enzymatic load with regard to the development of the process.

Regarding the amino nitrogen content, Figure 6c shows its evolution in the elaborated fish sauces during their fermentation time. Amino nitrogen is the chemical form of nitrogen when present as a primary amino group. This can be used as an indicator of food quality for fermented fish products, and it is usually related to their degree of fermentation [57]. The evolution of amino nitrogen presented the same trend as that of the total and formaldehyde nitrogen contents, where levels increased with fermentation time. These results suggest that the nitrogenous compounds were hydrolyzed into small fragments, probably composed of amino acids, as indicated by Klomklao et al. [9]. Regardless of its evolution, the final amino nitrogen content in the fish sauces produced after 20 days of fermentation was between 14.0 and 15.3 g N/L. According to the Thai Industrial Standard [61], amino nitrogen contents must be above 10 g/L, which means that the sauces produced in our study would comply with this regulation. The ammonia nitrogen content evolution of the fish sauce samples can be seen in Figure 6d. Its content rose in all the samples as the fermentation time increased, with the C and SUG samples exhibiting very similar behaviors.

Several authors have pointed out that the amount of ammonia nitrogen signifies the breakdown of soluble protein and peptides into free amino acids and volatile nitrogen [7,59,62]. Thus, the increment of ammonia nitrogen indicates the deamination or decomposition of nitrogenous compounds, as well as of the proteins in the fish and sea urchin associated to microbial activity and spoilage [9]. In all the fish sauces elaborated for our research, the final ammonium nitrogen contents were very low (0.7–0.8 g/L) in comparison to those reported by other studies. This low content together with that of TBV corroborates that no fermentation deviation and, therefore, fish sauce spoilage occurred in any of our sauces. On the other hand, greater ammonia nitrogen contents were observed in the control samples from the beginning of the fermentation process, which denotes a certain correlation between this content and that of TVB-N (Figure 4a). These results also suggest that ammonium nitrogen content could be related to the enzymatic activity and to the freshness of the fish used for the preparation.

### 3.6. Evolution of the Microbiological Count

**Figure 7:**
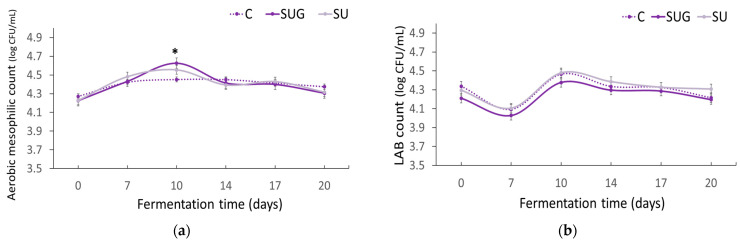
Evolution of the total viable count (log CFU/mL) of aerobic mesophilic (**a**) and LAB (**b**) in the fish sauces during their fermentation process. (C: control; SUG: sea urchin gonads; SU: whole sea urchin). Asterisks indicate the significant difference between the control samples (C) and the samples with sea urchin addition (SUG or SU) at each of the sampling time points (*p* < 0.05).

The evolution of the aerobic mesophilic and lactic acid bacteria (LAB) counts is shown in Figure 7. As can be seen, the initial counts of aerobic mesophilic and lactic acid bacteria remained within a range of 4.2–4.3 log CFU/mL, with no significant divergences between the different samples of fish sauce. With regard to the evolution of the aerobic mesophilic bacteria count during the fermentation process (Figure 7a), it should be noted that it presented an upward trend until day 7 in all of the cases, and from then on the trends corresponding to each specific sauce type, i.e., C, SUG, and SU, followed distinctive trends. Thus, in the control samples (C) there was a gradual growth of the mesophilic aerobic bacteria, which was more accentuated during the first 7 days and from then on it remained rather steady until day 14, when it exhibited a gradual and gentle downward trend until day 20. In contrast, the samples containing sea urchin (SUG and SU) grew continuously until reaching its maximum level on day 10 (4.6 and 4.55 for SUG and SU, respectively). This additional increase from day 7 to day 10 could be attributable to the starting of the stirring procedures of the preparation from day 7. Stirring favored micronization and greater contact and more thorough mixing of the ingredients in the formulation, in addition to providing a larger amount of oxygen that could enhance the growth of mesophilic aerobic bacteria. The decrease in aerobic mesophylls from day 10, mainly in the SUG and SU sauces, could be due to the extreme conditions regarding salt concentration, temperature, and, above all, pH. Likewise, this decrease could also be due to the absence of an adequate substrate for the development of these microorganisms and to the inhibition caused by certain products as suggested by other authors [3,11,34,35,57]. 

The incorporation of whole sea urchins with their shells implied the addition of aerobic mesophilic bacteria, which resulted in generally higher bacterial counts than those corresponding to the SUG samples. This phenomenon is of great interest, given that the SU samples contained a lower proportion of gonads.

The lactic acid bacterial counts (Figure 7b) showed similar growth and decline trends for the three sauces, with SUG samples exhibiting slightly lower counts than the control sauce. During the first 7 days of fermentation the number of LAB went down in all of the cases, probably due to the shock caused by the high salt concentration and the high temperature. However, once the bacteria adapted to the medium, and agitation favored their development, their number went up until reaching its maximum on day 10. Results revealed no significant differences between the three sauces (*p* > 0.05), although the presence of the shell seemed to favor the growth of lactic acid bacteria, so that the SU sample reached very similar maximum levels to those of the control sample. LAB converts glucose into organic acids that cause the pH to go down [6,63], hence the upward and downward trends of LAB counts were directly related to the evolution of the sauces’ pH value (Figure 1). A correlation between the development of aerobic mesophilic and lactic acid bacteria according to the evolution of nitrogenous compounds and total nitrogen (Figure 6) could be observed.

### 3.7. Quantitative Descriptive Analysis (QDA) of Sauces

The specific aromas and flavors of fish sauces are determined by the diverse compositions of their raw materials (fish species, ingredients), the fermentation conditions, and the fermentation process [64]. The combined action of all these factors results in the degradation of the proteins and lipids in the fish flesh via various biochemical metabolic pathways subject to the synergistic action of both the halophilic microorganisms and the enzymes that are present in the formulation [65].

**Figure 8:**
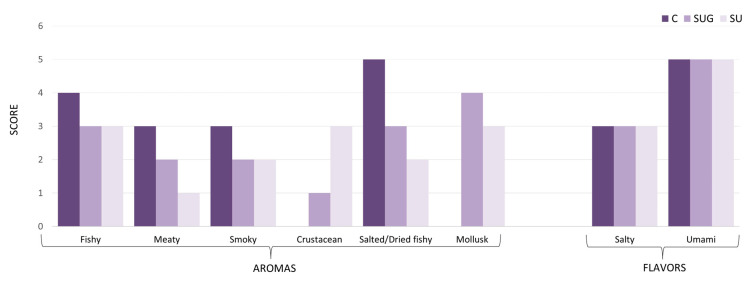
Sensorial characteristics of the three fish sauces base on the QDA (C: control; SUG: sea urchin gonads; SU: whole sea urchin). The intensity of each attribute was measured on a lineal, nonstructured scale from 0 (sensation not perceived) to 5 (maximum sensation).

Figure 8 shows the eight outstanding organoleptic characteristics of the fish sauce samples produced in this study based on their respective Quantitative Descriptive Analysis (QDA), where a panel composed of ten individuals has evaluated a total of ten attributes. According to this evaluation, it can be seen that their aromatic attributes, rather than their taste attributes, were more clearly affected by the addition of sea urchin to the formulation. Thus, all three types of sauces presented a similar intensity of umami and salty flavors, with no crustacean flavors standing out over the fish. Nonetheless, the usage of sea urchin resulted in an increment in the mollusk and crustacean aroma scores and lower meaty, fishy, smoky, and salted/dried fishy aroma scores. With regard to the tasters’ preferences, the sauces that contained sea urchin stood out for their particular aroma of mollusks and crustacea, but no order of preference between sea urchin sauces with or without shell could be established.

## 4. Conclusions

The influence of sea urchin addition on the development of the fermentation and the physiochemical characteristics of a variety of fish sauces mainly made from anchovies was studied. According to the results, the addition of sea urchin with and without its shell did not modify the fermentation process with respect to the control sauce despite the lower proportion of anchovies. The use of whole sea urchin, including its shell, implied a greater presence of aerobic mesophiles and lactic acid bacteria, which led to an increased protease activity and a greater production of total nitrogen, amino nitrogen, and formaldehyde nitrogen. Nevertheless, for pH, TMA, and TVB-N values, no deviation from traditional fermentation showed itself and, therefore, no spoilage of the fish sauce occurred as a consequence of the addition of sea urchin in any of the cases. Furthermore, the sensory evaluation of the urchin-added sauces was rather satisfactory, as they exhibited similar sensory characteristics to those of the control sauce, with the exception of their more intense crustacean and mollusk aromas. According to the data gathered from our research study, we could conclude that the addition of sea urchin to fish sauce traditional formulations may enhance the sensory qualities of the final products and specifically those corresponding to their aroma rather than flavor, as sea urchin contributes to obtain a sensory profile that exhibits more intense crustacean and mollusk aromas. In short, there is high added value to the final product that is obtained by including sea urchins in the preparation of traditional fish sauces.

## Data Availability

The data used to support the findings of this study can be made available by the corresponding author upon request.
